# Evaluation of conventional and response surface level optimisation of *n*-dodecane (*n*-C12) mineralisation by psychrotolerant strains isolated from pristine soil at Southern Victoria Island, Antarctica

**DOI:** 10.1186/s12934-018-0889-8

**Published:** 2018-03-17

**Authors:** Syahir Habib, Siti Aqlima Ahmad, Wan Lutfi Wan Johari, Mohd Yunus Abd Shukor, Siti Aisyah Alias, Khalilah Abdul Khalil, Nur Adeela Yasid

**Affiliations:** 1 Department of Biochemistry, Faculty of Biotechnology and Biomolecular Sciences, Universiti Putra Malaysia, 43400 Serdang, Selangor Malaysia; 2 Department of Environmental Sciences, Faculty of Environmental Studies, Universiti Putra Malaysia, 43400 Serdang, Selangor Malaysia; 3 Institute of Ocean and Earth Sciences, C308 Institute of Postgraduate Studies, University of Malaya, 50603 Kuala Lumpur, Malaysia; 4 Department of Biomolecular Sciences, Faculty of Applied Sciences, Universiti Teknologi MARA, 40450 Shah Alam, Selangor Malaysia

**Keywords:** Biodegradation, Diesel fuel, Psychrotroph, Response surface methodology, GC-FID analysis

## Abstract

**Background:**

Biodegradation of hydrocarbons in Antarctic soil has been reported to be achieved through the utilisation of indigenous cold-adapted microorganisms. Although numerous bacteria isolated from hydrocarbon-contaminated sites in Antarctica were able to demonstrate promising outcomes in utilising hydrocarbon components as their energy source, reports on the utilisation of hydrocarbons by strains isolated from pristine Antarctic soil are scarce. In the present work, two psychrotolerant strains isolated from Antarctic pristine soil with the competency to utilise diesel fuel as the sole carbon source were identified and optimised through conventional and response surface method.

**Results:**

Two potent hydrocarbon-degraders (ADL15 and ADL36) were identified via partial 16S rRNA gene sequence analysis, and revealed to be closely related to the genus *Pseudomonas* and *Rhodococcus* sp., respectively. Factors affecting diesel degradation such as temperature, hydrocarbon concentration, pH and salt tolerance were studied. Although strain ADL36 was able to withstand a higher concentration of diesel than strain ADL15, both strains showed similar optimal condition for the cell’s growth at pH 7.0 and 1.0% (w/v) NaCl at the conventional ‘one-factor-at-a-time’ level. Both strains were observed to be psychrotrophs with optimal temperatures of 20 °C. Qualitative and quantitative analysis were performed with a gas chromatograph equipped with a flame ionisation detector to measure the reduction of *n*-alkane components in diesel. In the pre-screening medium, strain ADL36 showed 83.75% of *n*-dodecane mineralisation while the reduction of *n*-dodecane by strain ADL15 was merely at 22.39%. The optimised condition for *n*-dodecane mineralisation predicted through response surface methodology enhanced the reduction of *n*-dodecane to 99.89 and 38.32% for strain ADL36 and strain ADL15, respectively.

**Conclusions:**

Strain ADL36 proves to be a better candidate for bioaugmentation operations on sites contaminated with aliphatic hydrocarbons especially in the Antarctic and other cold regions. The results obtained throughout strongly supports the use of RSM for medium optimisation.

## Background

Antarctica, once a pristine ice-covered landmass is being contaminated with pollutants, largely hydrocarbons. Petroleum hydrocarbons are widely used in Antarctic as human demands for heating, transportation and power generation [1]. One of the common hydrocarbon fuels used in the Antarctic region is diesel. Diesel oil, a complex combination of aliphatic and aromatic hydrocarbons is obtained from the middle-distillate, gas-oil fraction during fractional distillation of petroleum [2]. There is also the presence of branched-chain hydrocarbons and aromatic compounds, although in lesser amounts [3]. However, due to their immense production and use as fuels for transportation, contamination in the polar pristine soil are likely bound to occur [4, 5]. Oil spills and leakage from the underground storage tanks and pipelines are also likely to pollute the unfrozen soil active layer due to their low viscosity and mobile properties [6]. Aislabie et al. [7] reported that oil spills frequently occur near to the research and fuel stations where fuel storage and refueling of aircrafts and vehicles are heavily operated. The contamination can also cause risks for humans and other living organisms if diesel fuel reaches groundwater reservoirs and water bodies [8]. Besides, the freeze–thaw cycle and the lower temperatures in the Antarctic means that spilt fuel may persist longer, which may indicate that recovery of the Antarctic ecosystem, is slower than in temperate climates [9, 10]. These negative effects toward organism are of great concern, and techniques to remove the pollutants are strongly demanded.

Due to the remoteness from proper infrastructure for remediation of the Antarctic environment, bioremediation is acknowledged as the adequate approach to recuperate contaminated soils as it is simple, economical and causes lesser environmental impact [1, 11, 12]. Since the Antarctic Treaty outlaws the introduction of foreign organisms, microbes that are indigenous to the Antarctic soil are required for the application of bioremediation [13]. Besides, indigenous microbes that are able to degrade hydrocarbon contaminants are already adapted to the pollutant presence for growth and survival towards the extreme conditions exist in soils which are typically cold, dry, alkaline and low in nutrients during the summer seasons [14]. Several hydrocarbon-degrading bacteria that commonly isolated from polluted Antarctic soils are typically allocated to the genera *Acinetobacter*, *Pseudomonas*, *Rhodococcus* or *Sphingomonas* [1, 5]. These bacterial species are known to degrade both alkanes [15, 16] and/or aromatic hydrocarbons [13] aerobically.

Previously isolated bacteria able to utilise hydrocarbons are reported to depend strongly on the physical and medium factors [17, 18]. Therefore, an optimisation process must be done to achieve maximum hydrocarbons degradation. In the conventional approach, individual factors are optimised by changing one factor at a time while keeping other variables at a constant value. Although the ‘one-variable-at-a-time’ method can be implemented easily in helping the selection of optimum analysed parameters, it is meticulous, time-restraining and overlooks interactions among factors affecting a particular response. Contrariwise, statistical analysis such as response surface methodology (RSM) measures and interprets the responses obtained from a batch run while studying the interactions between the individual factors assessed. RSM is also more beneficial in conducting experiment as it reduced experimental time and runs [19]. Comparative analysis between the conventional and response surface method has been studied previously with the latter approach proved to be more successful in determining optimal point in studied parameters [20, 21].

The aim of this study was to isolate and identify the diesel-degrading bacteria from pristine soils collected from Scott Coast and Ross Island, Antarctica. Respective strains identified by 16S rRNA sequence analysis were then characterised by their biodegradative abilities and growth at optimised parameters. Degradation of *n*-dodecane was also estimated at two different level of optimisation approaches—conventional ‘one-factor-at-a-time’ and statistical method to ascertain the efficiency of both methods in optimising the mineralisation of dodecane.

## Methods

### Samples and media

Soil samples collected from pristine Antarctic areas were labelled as 1–43. The collected samples were kept in appropriate coolers (3–5 °C) during transportation from the Antarctic mainland to Malaysia. Diesel fuel used throughout the experiment was purchased from the local gas station (PETRONAS) in Selangor, Malaysia. Alkane analytical standard C_8_–C_20_ was purchased from Sigma-Aldrich, USA. For the isolation of hydrocarbon-degrading bacteria and growth optimisation, a standardised Bushnell-Haas (BH) broth was used [22]. The BH media is composed of (g/l): MgSO_4_ (0.2); CaCl_2_ (0.02); K_2_HPO_4_ (1.0); KH_2_PO_4_ (1.0); NH_4_NO_3_ (1.0) and FeCl_3_ (0.05); with the addition of 20 g bacteriological agar for solid media. The pH of the media was adjusted to 7.0 ± 0.2 at 25 °C before sterilisation. Diesel was the sole carbon source used in all experiments. Uninoculated sterile BH agar and broth were used as controls for confirmation of strain ability to utilise diesel as a sole carbon source.

### Strain isolation and screening

One gram of each soil sample was weighed and suspended in 10 ml of sterile saline solution (0.9% w/v NaCl), incubated at 10 °C on a shaking incubator at 160 rpm for 1 h, before serially diluted up to 10^7^. Aliquots (0.1 ml) of each dilution were spread onto the BH agar (BHA) supplemented with 0.5% (v/v) diesel fuel sterilised by filtration through 0.45-µm membrane filters (Lab-Firma), as a sole carbon source. The sterilised diesel fuel was added separately into the media before inoculation. Agar plates were incubated at 4 °C for 14 days. Isolates displaying distinct colonies were isolated and further purified via streak plate method. Pure isolates were then screened based on their hydrocarbon-degrading capacity. Screening was verified using the Hanson et al. [23] well assays test which based on the colour change of the redox indicator 2,6-dichlorophenolindophenol (DCPIP). The principle of the screening experiment lies in the electron transfer during the microbial oxidation of hydrocarbons, where O_2_ acts as the electron acceptor. Hence, the incorporation of electron acceptor such as DCPIP can ascertain the competency of the microorganism to utilise hydrocarbon substrate by observing the colour change of DCPIP from blue (oxidised) to colourless (reduced). Two sets of control were prepared to lack of substrate and inoculums respectively. Based on the degradation study, the best substrate degraders were selected and subjected to identification.

### Strain identification

Selected strains were identified based on biochemical and molecular approaches. For the biochemical test, the isolates were characterised by cell morphology, Gram-reaction, as well as oxidase and catalase production. For molecular identification, genomic DNA was extracted using GeneJET Genomic Purification Kit (Thermo Scientific, USA) according to the manufacturer’s procedure and protocol. Enzymatic lysis buffer was made of 20 mM Tris–HCl (pH 8.0), 2 mM EDTA and 1.2% Triton X-100 prior to extraction. 20 mg/ml of lysozyme was added to the lysis buffer immediately before the routine. The taxonomic analysis of selected strain was characterised through amplification of 16S rRNA gene using the following forward (domain-specific) and reverse universal primer with the sequence of 27F (5′-AGAGTTTGATCCTGGCTCAG-3′) and 1492R (5′-TACGGTTACCTTGTTACGACTT-3′), respectively. The polymerase chain reaction (PCR) amplification mixture consisted of 1 µl of DNA template, 1 µl of 5 mM forward and reverse primer each, 12.5 µl of 2X *Taq* Master Mix (Vivantis, USA) with the addition of 9.5 µl sterile deionised water for a final volume of 25 µl. PCR was performed under following conditions: initial denaturation at 94 °C for 3 min; 34 cycles of denaturation at 94 °C for 1 min, annealing at 58 °C for 1 min, extension at 72 °C for 2 min, and a final elongation at 72 °C for 10 min with incubation at 4 °C. The amplified PCR product was separated on 1% (w/v) agarose gel electrophoresis. The PCR products were purified using GeneJET Gel Extraction and DNA Cleanup Kit (Thermo Scientific, USA) prior to sequencing process using ABI 3730*xl* DNA Analyzer (Applied Biosystems, USA).

### Phylogenetic analysis

The 16S rRNA gene sequences of each purified strain were identified using BLASTn [24]. The alignment of the sequences was used for analysis under the neighbor-joining method [25] fitting to the distances of Jukes-Cantor model [26]. Phylogenetic tree was constructed using PHYLIP software v3.696 [J. Q. Felsenstein, PHYLIP—phylogeny inference package, version 3.696] (http://evolution.genetics.washington.edu/phylip.html). *Bacillus anthracis* strain ATCC 14578 and *E. coli* strain U5/41 were used as the outgroups in the cladogram for ADL15 and ADL36, respectively. The evolutionary distance along with the robustness of the inferred trees was calculated and evaluated by neighbor-joining method by 1000 bootstraps replicates [27]. The constructed tree was viewed using the TreeView v3.0 software.

### Conventional ‘one-factor-at-a-time’ (OFAT) optimisation

Optimisation of bacterial growth and degradation of diesel were carried out using conventional method based on selected factors: initial pH, salinity, substrate concentration and temperature. The effect of these factors was evaluated in liquid culture (50 ml of BH media in 250 ml conical flask shaken at 150 rpm). Investigated factors were optimised by maintaining all factors at a constant level for the customary scaling-up strategy. Each consequent factor was examined after considering the previously optimised parameters. The optimised pH was measured using three separate buffers: acetate buffer (pH 5–6), phosphate buffer (pH 6–7.5) and Tris–HCl buffer (pH 7–8). Tolerance to salt was examined by the addition of increasing amount of NaCl—0, 1, 2, 3, 4 and 5% (w/v) to the medium. The effect of diesel concentration was verified at 0.5, 1, 2, 3, 4 and 5% (v/v). Effect of temperature was evaluated at 10, 15, 20, 25 and 30 °C. Cell suspensions were prepared prior to the study. For cell suspension preparation, isolates were initially grown in 100 ml of nutrient broth for 48 h prior to cell cultivation. Culture grown in nutrient broth (20 °C, 150 rpm) were then centrifuged (5000×*g*, 10 min), washed with 1× phosphate-buffered saline (PBS: 137 mM NaCl, 2.7 mM KCl in 10 mM phosphate buffer, pH 7.4) solution and the cell density was adjusted to OD_600_ nm = 1. All assays which included inoculated controls were performed in triplicates.

### Bacterial growth and hydrocarbon biodegradation analysis

The bacterial abundance was determined through the colony counting technique on nutrient agar (NA) plates. Concentration of the colony-forming units was expressed by means of logarithmic notation using the average of plate count from the triplicate flasks at the same time. Growth and degradation studies were carried out concurrently. Following the incubation times, the liquid cultures of each flask were extracted with *n*-hexane (1:1 media to *n*-hexane) as a solvent to separate the cellular material. The final residual oil extracts were transferred to tared vials and kept at 4 °C. The qualitative and quantitative analysis of residual hydrocarbons in diesel occurring in the extracts were observed with a gas chromatograph (7890A, Agilent Technologies), equipped with a DB-5MS capillary column (30 m × 0.25 mm × 0.25 µm) and a flame ionisation detector (FID). Oven temperature was initiated at 80 °C, then ramped at 20 °C/min to 280 °C and held at this temperature for 2 min. The inlet was run in split mode at 240 °C with a 50:1 split ratio. Hydrogen was used as the carrier gas with flow rate at 30 ml/min. GC peaks were characterised by injecting the standards of *n*-alkane (C_8_–C_20_) under the same condition. *n*-dodecane was used as the indicator for biodegradative losses in quantitative analysis as this *n*-alkane has a melting point of − 9.6 °C. This permits the incubation of cultures at any temperature above 0 °C as compared to the more common *n*-hexadecane. *n*-hexadecane has a melting point of 18.2 °C that prevent this study to compare the mineralisation at low and moderate temperature [18]. The biodegradation of hydrocarbon compounds in diesel oil was expressed as the percentage of hydrocarbons degraded with regard to the amount of the residual fractions in the appropriate abiotic control samples. The reduction of each degraded hydrocarbon was evaluated by means of biodegradation efficiency (BE) using the following expression:

$${\text{BE}}\left( \% \right) = 100 - \left( {A_{\text{s}} \times 100/A_{\text{ac}} } \right)$$

where, *A*_s_ = total area of peak in sample, *A*_ac_ = total area peak in abiotic control, BE(%) = percentage of biodegradation efficiency.

### Statistical analysis

The factors determined conventionally were screened out using Plackett–Burman (PB) factorial design to ascertain the influential variables among numerous interactions between parameters. The experimental design was generated and analysed by using statistical software Design Expert 6. Each variable (pH, temperature, substrate concentration, NaCl concentration) was evaluated at two levels: maximum and minimum levels (+ 1, − 1). Viable plate count was analysed as response. Box-Wilson Central Composite Design (CCD) was fitted to characterise the nature of response surface in the favoured experimental region while identifying the optimal level of studied significant variables. The effect of each variable on bacterial growth and hydrocarbon degradation was studied at five different levels with the combination of two factorial points, two axial points and a sole central point (+ 2, + 1, 0, − 1, − 2) (Table 1). Percentage of *n*-dodecane mineralisation was evaluated as the response. The experimental response was fitted to a second-order polynomial regression model containing linear, quadratic and interaction coefficient to predict the optimal condition and the existence of interaction between significant variables. The mathematical model used to fit the outcome to the independent factors as in equation:

$$Y = \beta_{0} + \sum\limits_{i = 1}^{k} {\beta_{\text{i}} X_{i} } + \sum\limits_{i = 1}^{k} {\beta_{\text{ii}} X_{{i^{2} }} } + \sum\limits_{1 \le i \le j}^{k} {\beta_{\text{ij}} X_{i} X_{j} }$$

**Table 1:** Experimental range and levels of four selected variables for CCD optimisation in actual and coded factors

Factors	Name	Unit	Experimental value				
			− 2	− 1	0	+ 1	+ 2
*X*_*1*_	pH	–	5.75	6.50	7.25	8.00	8.75
*X*_*2*_	Temperature	°C	0.00	10.00	20.00	30.00	40.00
*X*_*3*_	Diesel concentration	% (v/v)	− 0.50^a^	1.00	2.50	4.00	5.50
*X*_*4*_	NaCl concentration	% (w/v)	− 1.00^a^	0.00	1.00	2.00	3.00

^a^Negative value will be assumed as zero in actual experiment

where, *Y* represents the predicted response; *X* are the independent factors that influence *Y*; *k* is the number of factors; $\beta_{0}$ is the constant term; $\beta_{\text{i}}$ is the *i* th linear coefficient; $\beta_{\text{ii}}$ is the *i* th quadratic coefficient and $\beta_{\text{ij}}$ is the *ij* th interaction coefficient whereas *i* and *j* = 1, 2, 3; and *i *≠ *j* are coefficients in the model. The significance of each coefficient in the equation was determined by Fischer’s *F* test and analysis of variance (*p* < 0.05).

## Results

### Isolation and identification of hydrocarbon degrading strain

After the enrichment of diesel as a sole carbon and energy source on BHA, prevalent colony types competent for a sustainable growth were taken from isolation plates and purified. Nine pure strains obtained were subjected to a screening test to assess the hydrocarbon degrading potential. The degrading capacity of the isolates was confirmed by the colour change of DCPIP from blue to colourless after incubation. Among the isolates tested, two sample were able to decolourise DCPIP in the presence of 0.5% (v/v) diesel within 24 h signifying a promising potential of substrates degradation. The isolates were labelled as isolates ADL15 and ADL36 and further subjected to characterisation.

**Fig. 1:**
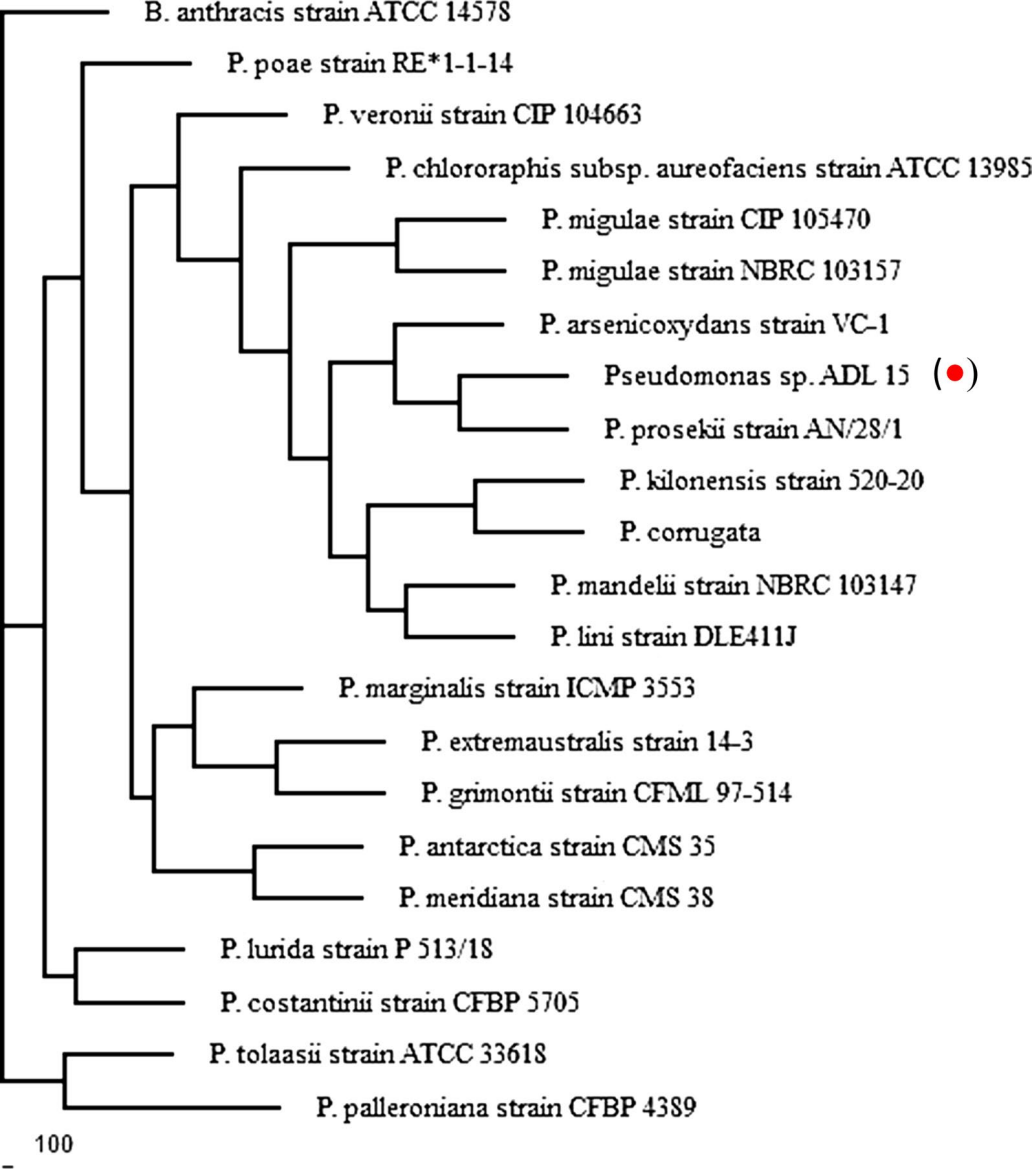
Phylogenetic analysis of 16S rRNA gene sequences using neighbour-joining method for ADL15 (filled red circle). (GenBank: KX812776)

**Fig. 2:**
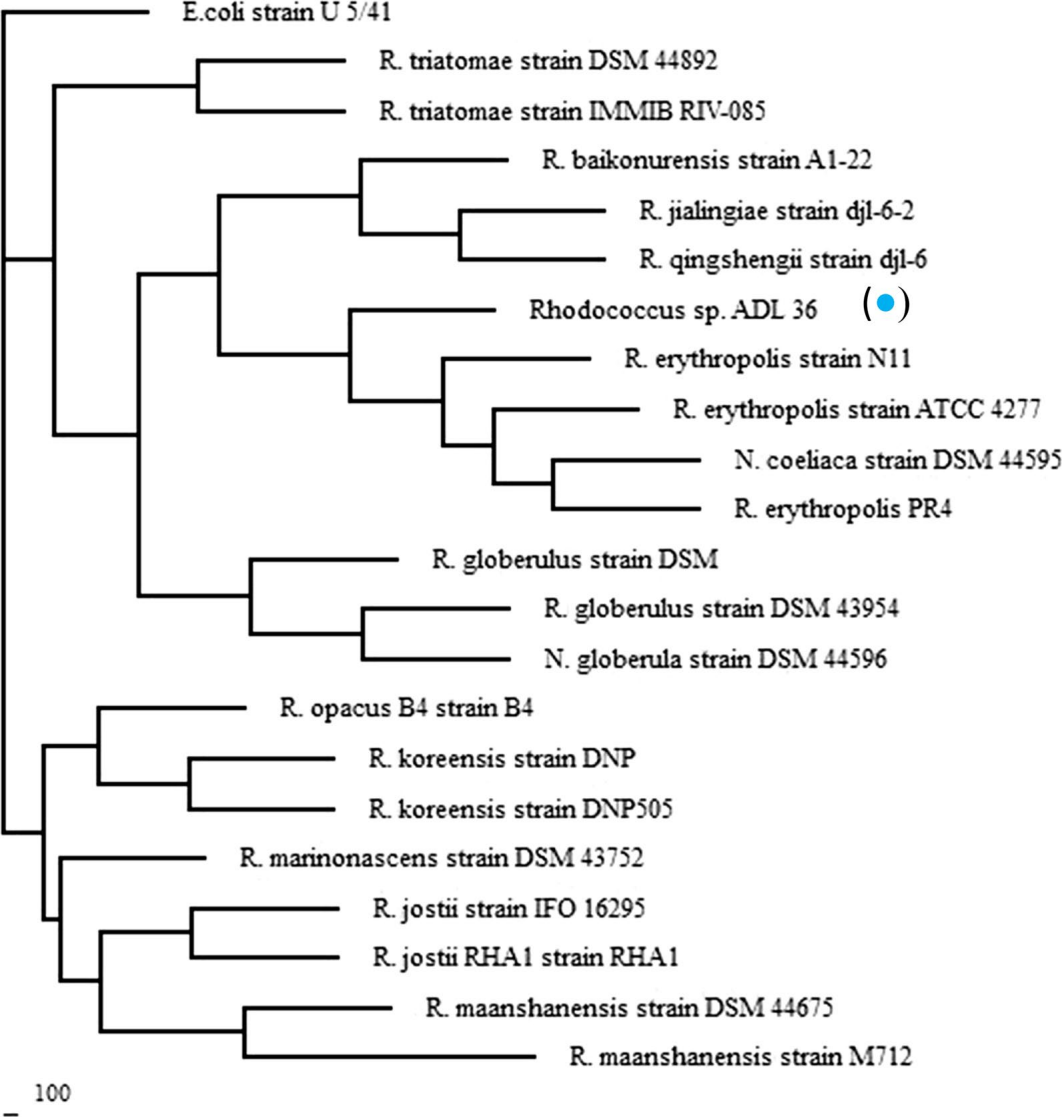
Phylogenetic analysis of 16S rRNA gene sequences using neighbour-joining method for ADL36 (filled blue circle). (GenBank: KX812777)

Isolate ADL15 is an aerobe, catalase, and oxidase producing, Gram-negative rod bacteria while ADL36 is an aerobe, catalase-positive, oxidase-negative, Gram-positive bacteria that exhibits the shape of short rods. Both strains formed raised, translucent white colonies on BHA while acquiring a raised and shiny colony with orange (Isolate ADL15) and yellowish (Isolate ADL36) pigmentation on nutrient agar. Identification of bacteria was supported by the 16S rRNA sequence analysis. Results from the BLASTn analysis showed that isolate ADL15 belonged to *Pseudomonas* genus with a high similarity percentage of (> 95%) whilst isolate ADL36 showed high similarity to *Rhodococcus* genus although displaying the highest similarity percentage (94%) to *Nocardia coeliaca* strain DSM 44595 and *Rhodococcus erythropolis* strain N11. It was noted that the 16S rRNA gene sequence of the former strain showed a surprising association with the genus *Rhodococcus* with a 99.9% similarity against the latter strain. Based on their partial 16S rRNA sequence, a phylogenetic tree for each strain was constructed, using *Bacillus anthracis* strain ATCC 14578 and *Escherichia coli* strain U 5/41 as the outgroup for ADL 15 and ADL 36 respectively (Figs. 1, 2).

Phylogenetic analysis based on these sequences showed that isolate ADL15 is closely related to *Pseudomonas prosekii* strain AN/28/1 with the nearest subclade of *Pseudomonas arsenicoxydans* strain VC-1. Isolate ADL15 was not assigned as *P. prosekii* species considering the low bootstrap value (< 75%). On the other hand, isolate ADL36 was descended into the well-supported subclade of the *R. erythropolis* strains.

### Optimisation using conventional ‘one-factor-at-a-time’ approach

**Fig. 3:**
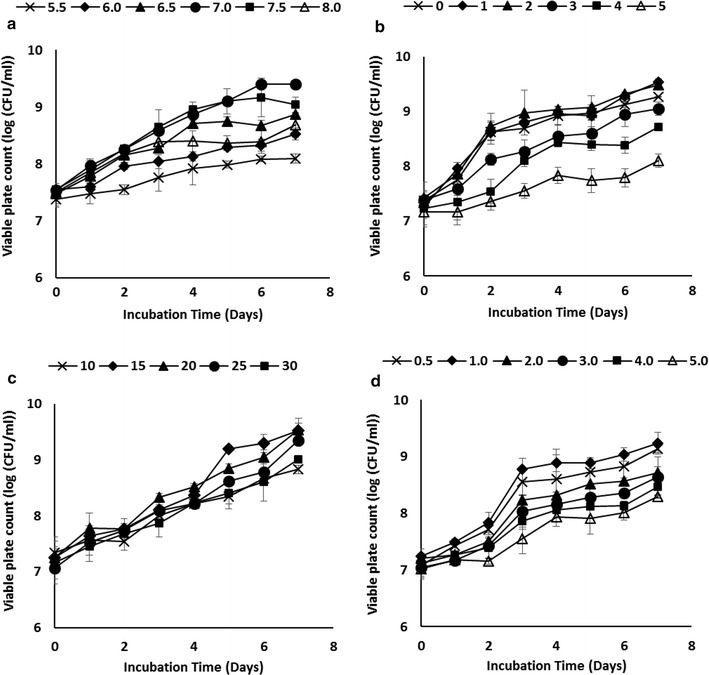
The effect of initial pH (**a**); salt concentration (**b**); temperature (**c**); and substrate concentration (**d**) on growth of strain ADL15 within 7 incubation days

**Fig. 4:**
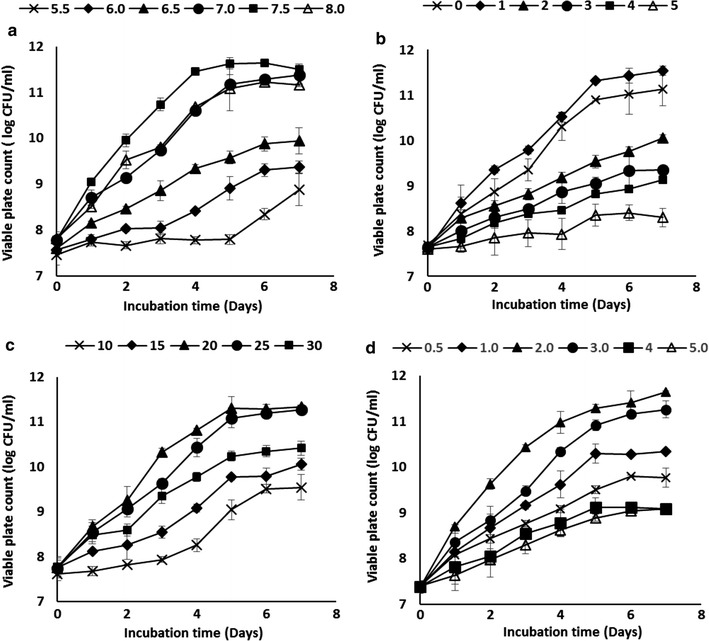
The effect of initial pH (**a**); salt concentration (**b**); temperature (**c**); and substrate concentration (**d**) on growth of strain ADL36 within 7 incubation days

The effect of selected factors—pH, NaCl concentration, temperature, and substrate concentration was initially assessed using the ‘one-factor-at-a-time’ method. Figure 3a shows the pH profile of *Pseudomonas* sp. ADL15. ADL15 grew best at pH 7.0 with no significant differences (*p *< 0.05) to any other pH tested. The lowest growth was observed at the lowest pH tested (5.5) after 7 days incubation. The salt tolerance of ADL15 was summarised in Fig. 3b. The results show that the best growth of isolate ADL15 was on the lower salt concentration [0, 1 and 2% (w/v)]. Sodium chloride concentration displayed a significant effect on viable plate count as increasing the salt concentration causes a lower cells growth. The influence of temperature on growth of ADL15 was presented in Fig. 3c. The strain shows an optimal temperature between 15 and 20 °C with no significant difference observed. The effect of substrate concentration on ADL15’s plate count was illustrated in Fig. 3d. As shown in the growth profile, the optimal substrate concentration was between 0.5 and 1.0% (v/v). Bacterial growth showed a gradual decrease as the diesel concentrations were increased. Figure 4a shows the influence of different initial pH on bacterial growth of *Rhodococcus* sp. strain ADL36. No significant differences in the plate count was observed at pH 7.0, 7.5 and 8.0 with the highest growth at pH 7.5 (log_10_ CFU/ml = 11.776). Strain ADL36 showed good adaptation on pH 6.5 but demonstrated a decreasing pattern towards the lower pH. Salinity studies showed that the optimal sodium salt concentration for ADL36 is at lower concentration (Fig. 4b). Similar to strain ADL15, an increase of salt amount in the cultured medium contributed to the reduction of cell’s growth. The temperature profile for the growth of strain ADL36 was showed in Fig. 4c. Strain ADL36 displayed a varied range of optimal temperature between 20 and 25 °C, as no significant difference was observed between this temperature values. The effect of diesel concentration on bacterial growth of strain ADL36 is shown in Fig. 4d. These results demonstrate that strain ADL36 exhibited a stronger adaptations towards higher diesel concentration than strain ADL15 with optimal growth between 2.0 and 3.0%. Each isolates exhibited a varied lag phase with noticeable increase in cell density at 4th and 5th day for strain ADL36 and ADL15, respectively. The growth curve of both isolates also demonstrated a short stationary phase after reaching the optimum growth value.

**Fig. 5:**
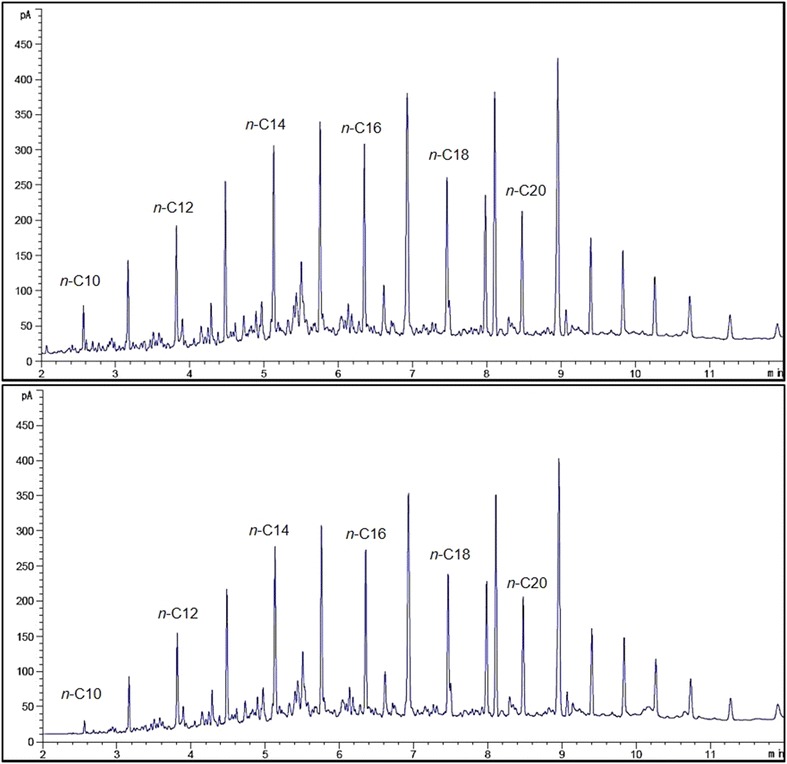
GC-FID profiles of hydrocarbons obtained from an optimised culture of strain ADL15 at 0 (top) and after 7 (bottom) days of incubation

**Fig. 6:**
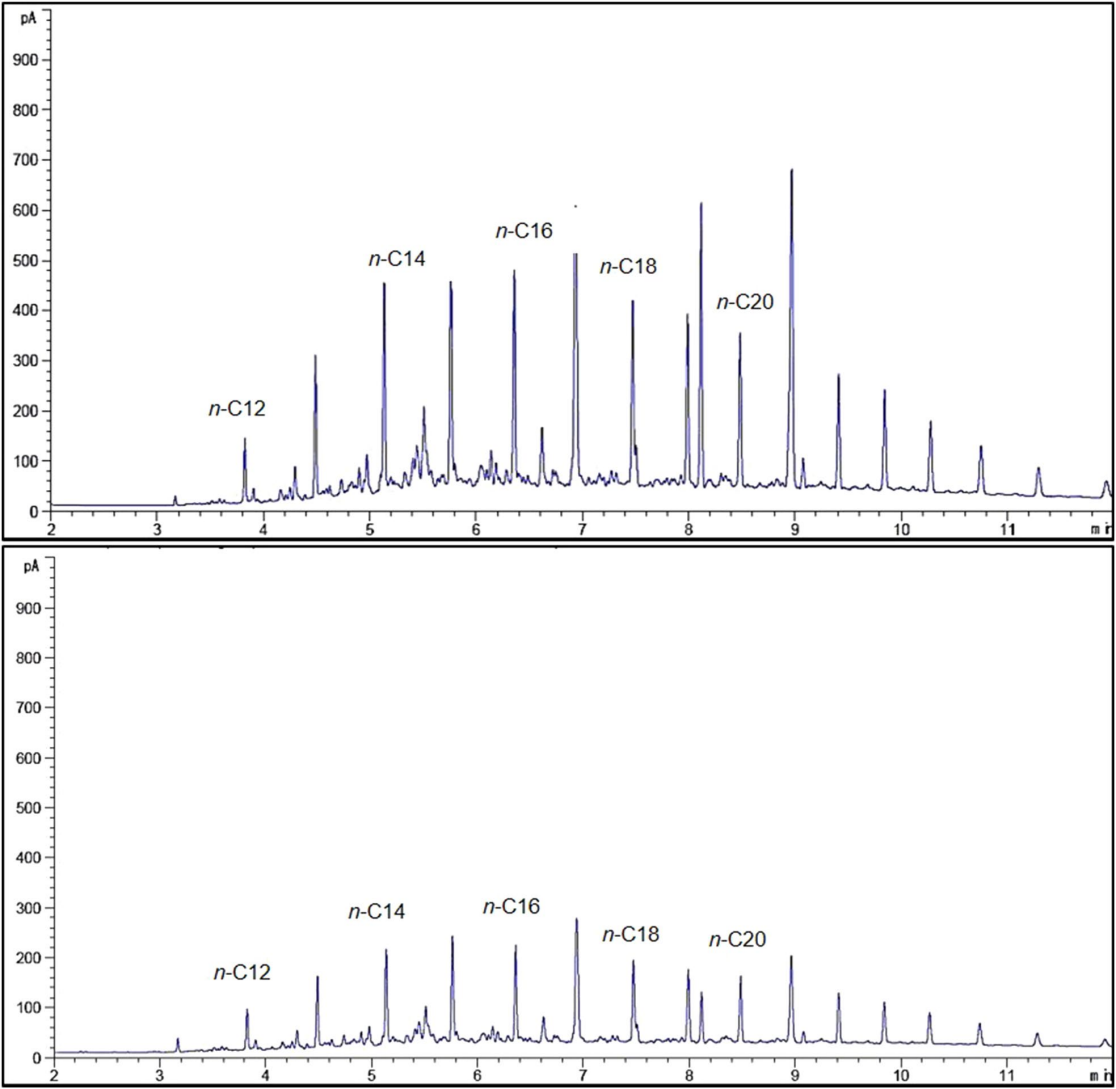
GC-FID profiles of hydrocarbons obtained from an optimised culture of strain ADL36 at 0 (top) and after 7 (bottom) days of incubation

A gas chromatographic analyses supported with flame ionisation detector (FID) was used to measure the mineralisation of *n*-alkanes by both strains in optimised media (Figs. 5, 6). Figure 5 shows that strain ADL15 tends to degrade shorter *n*-alkanes (*n*-C10, *n*-C11, *n*-C12) more efficiently than the middle-chain *n*-alkanes. However, the reduction of middle-chain *n*-alkanes was noticeably smaller than the shorter *n*-alkanes. GC-FID analysis for strain ADL36 revealed an utter contrast result to strain ADL15 with most of the recognisable *n*-alkanes significantly reduced. The reduction of the biodegradation indicator, *n*-dodecane by strain ADL15 and ADL36 after conventional optimisation was 22.39 and 83.75%, respectively.

### Optimisation using statistical approach

#### *Pseudomonas* sp. strain ADL15

The significant individual factors and interaction screened during Plackett–Burman design were brought into the central composite design with the percentage of *n*-dodecane mineralisation as the output response. Table 2 shows the experimental designs used in RSM studies by using four independent variables with six centre point showing both observed and predicted values for *n*-dodecane mineralisation (%).

**Table 2:** Experimental CCD on *n*-dodecane degradation by strain ADL15 with six centre points showing observed and predicted values

Run order	*X*_*1*_	*X*_*2*_	*X*_*3*_	*X*_*4*_	Dodecane mineralisation (%)	
					Experimental value	Predicted value
1	7.25	40.00	2.50	1.00	0.83	4.86
2	8.00	30.00	4.00	2.00	0.71	0.82
3	7.25	20.00	2.50	− 1.00	34.77	32.88
4	7.25	20.00	2.50	1.00	33.34	30.73
5	7.25	20.00	2.50	1.00	32.01	30.73
6	8.75	20.00	2.50	1.00	17.27	16.70
7	8.00	10.00	1.00	0.00	14.42	12.79
8	8.00	10.00	4.00	2.00	0.04	0.99
9	8.00	30.00	1.00	0.00	26.50	24.43
10	7.25	20.00	2.50	1.00	31.67	30.73
11	7.25	20.00	2.50	1.00	30.45	30.73
12	7.25	0.00	2.50	1.00	0.01	− 3.17
13	5.75	20.00	2.50	1.00	20.00	21.42
14	6.50	10.00	4.00	0.00	4.34	6.22
15	6.50	30.00	1.00	0.00	29.81	26.77
16	8.00	30.00	4.00	0.00	19.00	21.46
17	6.50	10.00	4.00	2.00	0.06	3.37
18	7.25	20.00	2.50	1.00	30.12	30.73
19	6.50	10.00	1.00	0.00	17.46	18.57
20	7.25	20.00	5.50	1.00	1.71	− 4.94
21	6.50	30.00	4.00	0.00	19.27	19.77
22	6.50	30.00	4.00	2.00	0.23	− 0.23
23	8.00	30.00	1.00	2.00	4.89	0.92
24	8.00	10.00	1.00	2.00	5.70	6.43
25	6.50	30.00	1.00	2.00	6.35	3.89
26	8.00	10.00	4.00	0.00	0.80	4.48
27	7.25	20.00	2.50	3.00	3.78	6.51
28	7.25	20.00	2.50	1.00	26.83	30.73
29	7.25	20.00	− 0.50	1.00	0.00	7.50
30	6.50	10.00	1.00	2.00	17.38	12.84

CCD data are shown as mean ± SD, n = 3

*X*_*1*_: pH; *X*_*2*_: temperature (°C); *X*_*3*_: diesel concentration (% v/v); *X*_*4*_: NaCl concentration (% w/v)

A maximum *n*-dodecane mineralisation of 34.77% was achieved at pH 7.25, 20 °C, 2.5% (v/v) diesel without any presence of NaCl (run 3, Table 2). The multiple regression analysis of the observed responses resulted in the quadratic equation of:

$$\begin{aligned} Y & = +\, 30.73 - 1.18{\text{A }} + 2.01B - 3.11C \hfill \\ & \quad - 6.59D - 2.92A^{2} - 7.47B^{2} - 7.36C^{2} \hfill \\ & \quad - 2.76D^{2} + 0.86AB + 1.01AC - 0.16AD \hfill \\ & \quad + 1.34BC - 4.29BD + 0.72CD \hfill \\ \end{aligned}$$

**Fig. 7:**
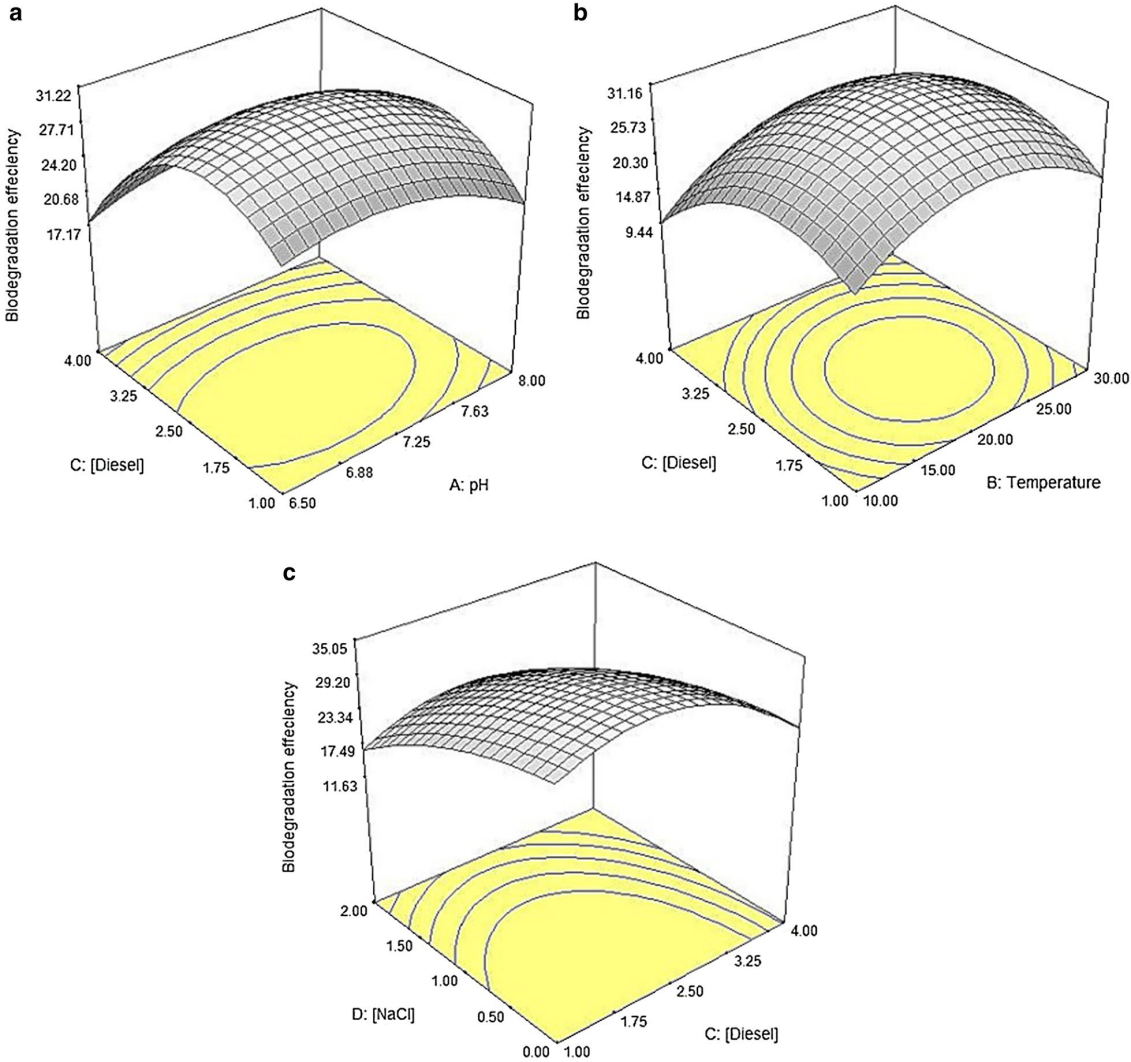
Response surface plots showing the significant interaction between diesel concentration and pH (**a**); temperature (**b**) and NaCl concentration (**c**) affecting the *n*-dodecane mineralisation (%) by strain ADL15

Table 3 shows the ANOVA for response surface for the percentage of *n*-dodecane mineralisation by strain ADL15. From Table 3, it can be observed that three linear terms (*B*, *C*, *D*), four squared terms (*A*^2^, *B*^2^, *C*^2^, *D*^2^), and a quadratic term (*BD*) of the model were significant to the response which indicates that the two most significant factors were the temperature (*B*) and amount of salt (*D*). Figure 7 describes the interaction between diesel concentrations with other individual factors on the percentage of *n*-dodecane mineralisation. The analysis of the 3D interactive plots showed that decreasing the NaCl concentration promotes a higher mineralisation of *n*-dodecane (Fig. 7c), while a higher degradation of dodecane occurred within a wide range of pH (with respect to amount of diesel used) (Fig. 7a) and intermediate temperature value (Fig. 7b).

**Table 3:** ANOVA for *n*-dodecane mineralisation by strain ADL15 in CCD

Source	Sum of squares	DF	Mean square	*F* value	Prob > *F* (*p* value)
Model	4455.95	14	318.28	18.28	< 0.0001*
*A*	33.41	1	33.41	1.92	0.1862**
*B*	96.80	1	96.80	5.56	0.0324*
*C*	232.13	1	232.13	13.33	0.0024*
*D*	1042.93	1	1042.93	59.91	< 0.0001*
*A* ^*2*^	233.71	1	233.71	13.42	0.0023*
*B* ^*2*^	1531.72	1	1531.72	87.99	< 0.0001*
*C* ^*2*^	1486.93	1	1486.93	85.41	< 0.0001*
*D* ^*2*^	208.81	1	208.81	11.99	0.0035*
*AB*	11.79	1	11.79	0.68	0.4235**
*AC*	16.27	1	16.27	0.93	0.3491**
*AD*	0.40	1	0.40	0.023	0.8816**
*BC*	28.58	1	28.58	1.64	0.2195**
*BD*	293.97	1	293.97	16.89	0.0009*
*CD*	8.26	1	8.26	0.47	0.5014**
Residual	261.13	15	17.41		
Lack of fit	236.16	10	23.62	4.73	0.0502**
Pure error	24.98	5	5.00		
Cor total	4717.08	29			
Standard deviation	4.17		R^2^		0.9446
Mean	14.32		Adjusted R^2^		0.8930
C.V.	29.13		Predicted R^2^		0.7040
PRESS	1396.23		Adeq precision		12.819

*A*: pH; *B*: temperature (°C); *C*: diesel concentration (% v/v); *D*: NaCl concentration (% w/v)

* Significant; ** not significant

**Fig. 8:**
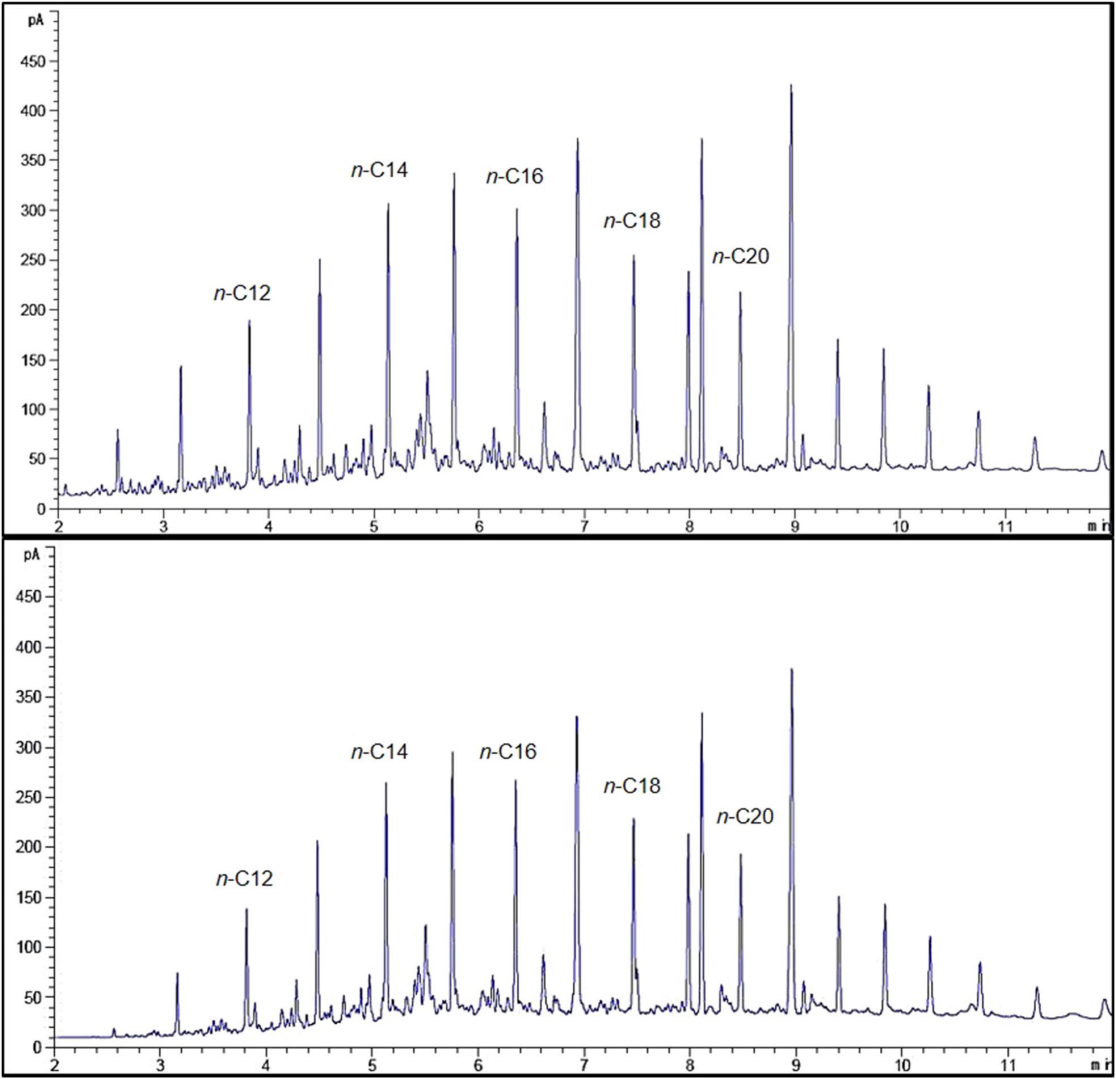
GC-FID profile of hydrocarbons obtained from the RSM-optimised ADL15 culture at 0 (top) and after 7 (bottom) days of incubation

The predicted optimum conditions were validated using modified BH media in accordance with the predicted model values. The percentage of *n*-dodecane mineralisation was significantly higher than the predicted value (36.33%) at 38.32%. The CCD-optimised media was proved to be more efficient compared to the conventional optimisation (22.39%). Figure 8 shows the GC-FID profile of the residual hydrocarbons in CCD-optimised media inoculated with strain ADL15.

#### *Rhodococcus* sp. strain ADL36

All parameters tested in Plackett-Burman design—pH, temperature, diesel concentration and NaCl concentration—have significantly affected the mineralisation of *n*-dodecane. The CCD design of RSM experiments using four independent factors with the experimental and predicted values for *n*-dodecane mineralisation was presented in Table 4.

**Table 4:** Experimental CCD on dodecane degradation by strain ADL36 with six centre points showing observed and predicted values

Run order	*X*_*1*_	*X*_*2*_	*X*_*3*_	*X*_*4*_	Dodecane mineralisation (%)	
					Experimental value	Predicted value
1	7.25	40.00	2.50	1.00	18.26	25.81
2	8.00	30.00	4.00	2.00	17.05	18.66
3	7.25	20.00	2.50	− 1.00	95.67	91.93
4	7.25	20.00	2.50	1.00	93.34	88.76
5	7.25	20.00	2.50	1.00	93.01	88.76
6	8.75	20.00	2.50	1.00	37.27	41.80
7	8.00	10.00	1.00	0.00	14.16	15.72
8	8.00	10.00	4.00	2.00	3.61	6.12
9	8.00	30.00	1.00	0.00	57.00	51.69
10	7.25	20.00	2.50	1.00	90.12	88.76
11	7.25	20.00	2.50	1.00	87.67	88.76
12	7.25	0.00	2.50	1.00	0.70	− 6.23
13	5.75	20.00	2.50	1.00	70.00	66.09
14	6.50	10.00	4.00	0.00	34.34	43.74
15	6.50	30.00	1.00	0.00	69.27	67.24
16	8.00	30.00	4.00	0.00	71.50	72.04
17	6.50	10.00	4.00	2.00	10.63	14.85
18	7.25	20.00	2.50	1.00	87.45	88.76
19	6.50	10.00	1.00	0.00	50.46	47.75
20	7.25	20.00	5.50	1.00	21.71	10.11
21	6.50	30.00	4.00	0.00	76.81	79.80
22	6.50	30.00	4.00	2.00	12.01	10.92
23	8.00	30.00	1.00	2.00	25.70	16.78
24	8.00	10.00	1.00	2.00	24.89	20.80
25	6.50	30.00	1.00	2.00	20.35	16.83
26	8.00	10.00	4.00	0.00	17.09	19.50
27	7.25	20.00	2.50	3.00	23.78	28.13
28	7.25	20.00	2.50	1.00	81.00	88.76
29	7.25	20.00	− 0.50	1.00	0.00	12.23
30	6.50	10.00	1.00	2.00	37.38	37.32

CCD data are expressed as mean ± SD, n = 3

*X*_*1*_: pH; *X*_*2*_: Temperature (°C); *X*_*3*_: Diesel concentration (% v/v); *X*_*4*_: NaCl concentration (% w/v)

The maximum degradation of *n*-dodecane was observed at run 3 (95.67%) with the medium condition of pH 7.25, 20 °C and 2.5% (v/v) diesel, as well as total absence of NaCl, similar to strain ADL15. The result was subjected to multiple regression analysis with consequent second-order polynomial equation in terms of coded level representing dodecane mineralisation (*Y*), respectively:

$$\begin{aligned} Y & = +\, 88.76 - 6.07 {\text{A }} + 8.01B - 0.53C \hfill \\ & \quad - 15.95D - 8.70A^{2} - 19.7 4B^{2} - 19.40C^{2} \hfill \\ & \quad - 7.1 8D^{2} + 4.12AB + 1.95AC + 3.88AD \hfill \\ & \quad + 4.14BC - 10.00BD - 4.6 2CD \hfill \\ \end{aligned}$$

**Fig. 9:**
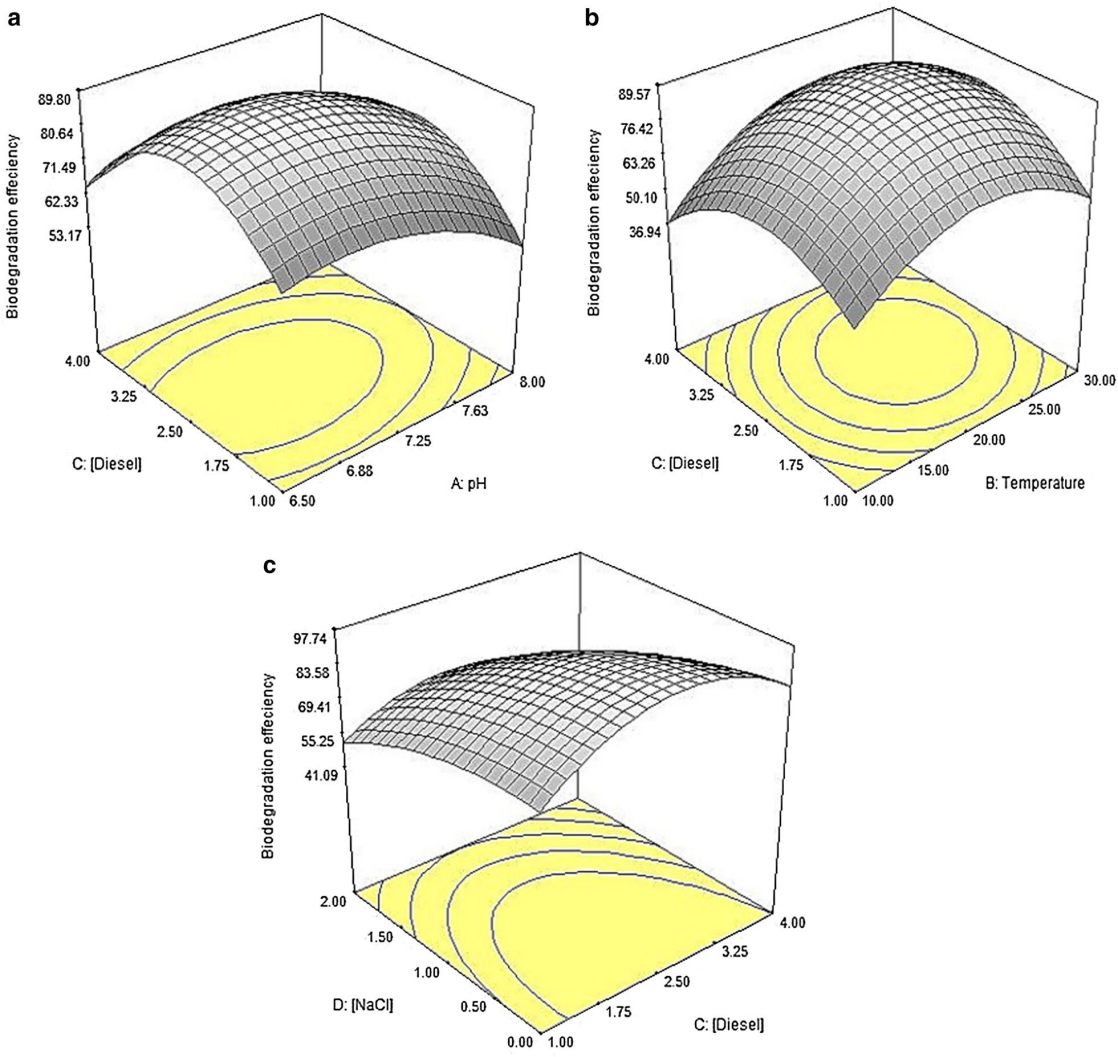
The 3D response surface plots showing the interaction between diesel concentration and pH (**a**); temperature (**b**) and NaCl concentration (**c**) affecting the *n*-dodecane mineralisation (%) by strain ADL36

Table 5 shows the ANOVA for response surface for the percentage of *n*-dodecane mineralisation by strain ADL36. It can be noted that three linear terms (*A*, *B*, *D*), four squared terms (*A*^2^, *B*^2^, *C*^2^, *D*^2^), and four quadratic terms (*AB, BC, BD, CD*) of the model were significant to the response. Similar to ADL15, the analysis of the 3D response curves and contours showed that a higher degradation of dodecane occurs within a wide range of pH (with respect to amount of diesel used) (Fig. 9a) and intermediate temperature value (Fig. 9b), while decreasing the NaCl concentration promotes a higher mineralisation of *n*-dodecane (Fig. 9c).

**Table 5:** ANOVA for dodecane mineralisation by strain ADL36 in CCD

Source	Sum of squares	DF	Mean square	*F* value	Prob > *F* (*p* value)
Model	30,222.84	14	2158.77	38.35	< 0.0001*
*A*	884.65	1	884.65	15.72	0.0012*
*B*	1540.05	1	1540.05	27.36	0.0001*
*C*	6.76	1	6.76	0.12	0.7337**
*D*	6105.04	1	6105.04	108.46	< 0.0001*
*A* ^*2*^	2078.28	1	2078.28	36.92	< 0.0001*
*B* ^*2*^	10,690.90	1	10,690.90	189.94	< 0.0001*
*C* ^*2*^	10,321.36	1	10321.36	183.37	< 0.0001*
*D* ^*2*^	1414.90	1	1414.90	25.14	0.0002*
*AB*	271.25	1	271.25	4.82	0.0443*
*AC*	60.76	1	60.76	1.08	0.3153**
*AD*	240.30	1	240.30	4.27	0.0565**
*BC*	274.49	1	274.49	4.88	0.0432*
*BD*	1598.79	1	1598.79	28.40	< 0.0001*
*CD*	340.93	1	340.93	6.06	0.0265*
Residual	844.30	15	56.29		
Lack of fit	740.26	10	74.03	3.56	0.0869**
Pure error	104.04	5			
Cor total	31,067.13	29			
Standard deviation	7.50		R^2^		0.9728
Mean	44.74		Adjusted R^2^		0.9475
C.V.	16.77		Predicted R^2^		0.8579
PRESS	4413.71		Adeq precision		18.503

*A*: pH; *B*: Temperature (°C); *C*: Diesel concentration (% v/v); *D*: NaCl concentration (% w/v)

* Significant; ** not significant

**Fig. 10:**
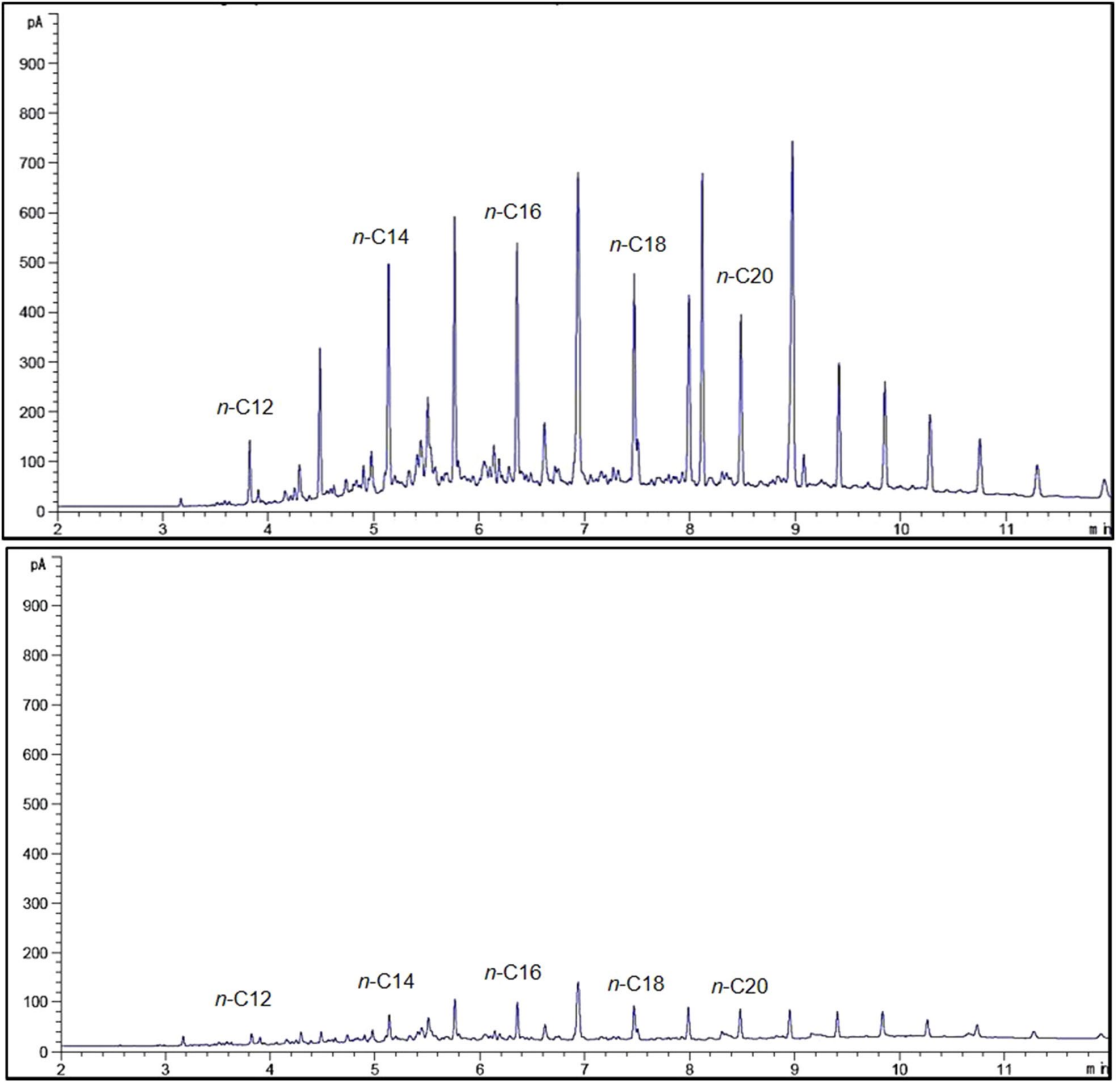
GC-FID profile of residual hydrocarbons obtained from the RSM-optimised culture at 0 (top) and after 7 (bottom) days of incubation with inoculation of strain ADL36

The optimum conditions predicted (desirability of 1, maximum degradation of 100) via RSM were validated by modifying the BH media in accord to the predicted values. Although significantly lower mineralisation of dodecane was achieved at 99.89%, the predicted medium was more efficient than the conventional-optimised medium (83.75%). Figure 10 shows the GC-FID profile of the hydrocarbons obtained in CCD-optimised media inoculated with strain ADL36 at 0 (control) and after 7 incubation days.

## Discussion

Antarctica proves to be one of the extreme environments to date due to their extreme cold and dryness, exceptionally strong winds and harsh storms. In spite of this, several life forms including microbial community have found their way to survive in this extreme condition due to their unique adaptation ability. Increased human activities such as scientific research, tourism and expedition led to a possible risk of pollution such as hydrocarbons. Fuel spills on soil raise the levels of soil organic carbon and may either serve as substrates for microbial growth or give off toxic effect to bacterial growth and activity [28]. Availability of oil contaminants in the environment to microorganisms influences the rate of biodegradation. Hydrocarbon compounds tend to bind to soil particles, making them harder to be degraded and removed [29]. Perry [30] reported that the susceptibility of hydrocarbons to microbial degradation could be arranged in decreasing order from the linear alkanes, branched alkanes, small aromatics, and cyclic alkanes to the low molecular weight polycyclic aromatic hydrocarbons (PAHs). Certain high molecular weight PAHs was also reported to be impossible to be broken down at all [31]. Albeit PAHs are the minor component of normal crude, they are among the most toxic to both plants and animals. Bacteria are most likely to degrade PAHs into biomass, carbon dioxide, and water. However, the initial insertion of oxygen molecule via dioxygenase enzymes are necessary for the process to take place [32].

As the Antarctic Treaty prohibits the introduction of non-Antarctic organisms, indigenous microbial population to the Antarctic were demanded for bioremediating hydrocarbon polluted soils. Strains ADL15 and ADL36 are two psychrotrophic strains isolated from soil collected from a pristine area in Cape Ross (76°43′54.70″S, 163°0′32.20″E) and Cape Royds (77°33′9.70″S, 166°9′30.60″E) respectively. The success in isolating a bacterial species with hydrocarbon-degrading ability from a pristine soil implies that hydrocarbon-degrading bacteria are components of the natural Antarctic microbiota prior to any possible contaminations.

Phylogenetic tree constructed using 20 nearest 16S rRNA gene sequence revealed that strain ADL15 is well-clustered into the *Pseudomonas* genus. Previous reports have shown that *Pseudomonas* sp. isolated from Antarctic soil manage to mineralise a range of hydrocarbon compounds such as aromatic compounds [13] and diesel fuel [16]. Ma et al. [33] also reported that several strains from *Pseudomonas* genus could degrade PAHs, which have low susceptibility towards microbial degradation. Strain ADL15 was clustered closely to the *P. prosekii* strain AN/28/1 and *Pseudomonas arsenicoxydans* strain VC-1. Records on hydrocarbon degradation by both species have yet to be reported, albeit the former has been reported as a novel psychrotrophic strain isolated from Antarctica with reported growth between 5 and 30 °C [34].

Another group of bacteria that are widely reported as cold-tolerant hydrocarbon degraders is *Rhodococcus* sp. [15, 35] due to their remarkable features; wide range of alkane compounds degradation and induction styles likely due to the presence of multiple alkane hydroxylase systems [36–38], production of biosurfactants [39, 40] and provide cell surface hydrophobicity [41]. These combined features together with their tolerance towards the cold environment have made them an effective tool for Antarctic soil remediation. Strain ADL36 fell under the subclade closely related to three strains of *R. erythropolis* and *Nocardia coeliaca*. Among the three related strains, *R. erythropolis* strain PR4 has been extensively studied as it is known for utilising ranges of hydrocarbon products as their carbon and energy sources [42–44]. The sequence of the strain’s genome also was determined by the presence of three plasmids harboured in the cells [45] while the metabolic responses of the strain growth on diesel oil and other hydrocarbons were studied [46]. On the other hand, *R. erythropolis* strain ATCC 4277 can synthesise a long-chain secondary alcohol dehydrogenase, which involves as part of the hydrocarbon metabolism in most hydrocarbon degraders [47].

From the results obtained in the physiological characterisation using ‘one-factor-at-a-time’ approach, strain ADL15 preferred a completely neutral pH for growth. Generally, *Pseudomonas* genus is capable of having an optimal growth at pH 6–8 and this might be due to the different adaptation towards each strain that has been isolated and investigated previously. On the other hand, strain ADL36 proved to be good in tolerance towards a more alkaline condition as optimal growth occurred between 6.5 and 7.5. Similar optimal pH for cold-tolerant *Rhodococcus* strain has been reported [48, 49]. Both strains demonstrate a clear affinity towards phosphate buffer (pH 6.0–7.5) in the overlapping buffer systems. The presence of phosphorus element in the buffer may serve either as a structural or functional role towards the cells. The strains’ intolerance to salt was observed to be skewed to the lower concentration. Although a high tolerance towards salt provides an advantage for bioremediating hydrocarbon contaminants in coastal and marine areas [50], both strains could be proposed to be implemented for bioremediation in normal soil.

The effects of initial hydrocarbon concentration on bacterial growth have been reported using a low hydrocarbon concentration [5, 18]. The use of lower concentration of substrate in most experiment is due to the toxic effects of the hydrocarbon components to the bacterial membrane. Previous studies have reported on *Pseudomonas* isolates with similar adaptability to a lower concentration of diesel/hydrocarbons [13, 35]. However, Shukor et al. [16] had successfully characterised an Antarctic *Pseudomonas* diesel-degrading strain that can optimally grow at 3.5% (v/v) diesel. Then again, ecotoxicological effects of petroleum to the bacterial strain could be determined by quantitative and qualitative differences in hydrocarbon content of petroleum products [51]. For that reason, the smaller growth of strain ADL15 in diesel-supplemented media could be owed to the low susceptibility attack of most *Pseudomonas* strain to the alkane-rich diesel fuel as they favour simpler aromatics compounds as their carbon source [13]. Diesel fuel proved to be a good energy and carbon source for strain ADL36 as *Rhodococcus* group widely known for utilising a range of alkanes for their energy sources [15]. Similar results on alkane-degrading Antarctic *Rhodococcus* also have been reported in previous experiments [5, 15]. Although strain ADL15 showed a smaller growth and alkane degradation compared to strain ADL36 in overall data, the former strain showed great adaptability towards diesel. This supports the observation from Atlas and Bartha [52] in attributing the members of *Pseudomonas* genera as the *r*-strategist, in which organism was promptly growing while adapting quickly to the presence of pollutants as their energy source. The stable growth of strain ADL36 could be explained by the k-strategy [52] where the *Rhodococcus* genera are known to reproduce at a slower rate while retaining a stable environment where new progeny is produced with a higher chance of survival and persistence.

Temperature is one of the most important factors in determining the success of biodegradation. Temperature affects the rate of microbial hydrocarbon degradation partially due to the physical nature of the spilt oil itself. As the viscosity of spilled oil increases, the volatilisation of short-alkanes is hindered, thus increasing their microbial toxicity and water solubility hence prolonging the onset of biodegradation [53]. Since the rate of reaction is thought to obey the Arrhenius relationship, biodegradation will decrease as temperature decreases, which is a con to the cold Antarctic environment. Due to these, bioremediation treatments are usually done and proposed in the summertime, with higher temperature, unfrozen soils and water is more accessible [54]. Both strains were not psychrophilic but psychrotolerant indeed, as the optimal temperature for bacterial growth > 15 °C but able to grow at a temperature near 0 °C [55]. The result is not surprising as psychrotolerant is a more prevalent group rather than psychrophilic due to the customary period of soils collection during the summertime, where the temperature of surface soils may reach up to 20 °C [56].

The data and reports regarding the mineralisation of diesel or specific hydrocarbon by Antarctic strains using statistical analyses hitherto were practically unavailable. However, the outcomes yielded by this study can still be compared with recent findings in term of dodecane mineralisation. Bej et al. [15] reported two *Rhodococcus* species isolated from Antarctica managed to mineralise dodecane up to 30% within a 15 day incubation period. The observed low dodecane mineralisation might be due to the lower temperature used for incubation and a smaller value of starting inoculum (OD_450_ nm = 0.35). The higher starting inoculum size used in this study must be highlighted. The bacterial inoculum was taken from an exponential phase culture of nutrient broth, suspended in 1× PBS solution and placed into a fresh BH medium. The purpose of using a high inoculum size (exponential phase) is to reduce the lag phase occurring in the new media (BH media) hence accelerating the rate of degradation. However, starting inoculum that is too high may disturb the synthesis of new replicate cells due to the overcrowded condition thus lead to cell toxicity and postpone the degradation of substrate. It must also be noted that the occurrence of exponential phase is dependent on the bacterial culture itself, which is different from one to another. Meanwhile, Whyte et al. [57] reported a comparable study of dodecane mineralisation at 20 °C using an Antarctic *Rhodococcus* strain, Q15. Strain Q15 was able to degrade 30% of dodecane while ADL36 was competent to utilise 96% dodecane within a 6-day incubation time, which is 2.2-fold higher than the former strain (data not shown). The contrasting results between these two strains could lie in the distinction in the culturing method in basal media. Mineralisation of dodecane by strain Q15 was assessed through 96-well plates without any shaking condition whereas strain ADL36 was cultured as described in Materials and methods. A shaking condition greatly affected the aeration of oxygen in cultured media as oxygen plays a significant role in determining the rate of hydrocarbon degradation [58–60]. With regards to the genus *Pseudomonas*, two psychrotrophic *Pseudomonas* strains able to mineralise dodecane was reported [61]. In the catabolic study, strain B17 and B18 were capable to utilise 17.4 and 15.6% of dodecane, respectively at 25 °C. Although the results obtained in the present study was significantly higher at 38.32%, the starting inoculum size of each strain from the former study was lower at OD_600_ nm = 0.025 than the present study (OD_600_ nm = 1) that could significantly affect the initial rate of dodecane mineralisation. Last but not least, a biostimulation experiment via microcosm conducted by Mohn and Stewart [4] accomplished to reduce up to 60% dodecane in soil at 7 °C. The study suggested that both ammonium (nitrogen source) and phosphate play major roles in biostimulation of dodecane in cold soil. This hypothesis holds true in this study, where ammonium and phosphate were used as the major composition in the culture media in the form of NH_4_NO_3_ (ammonium) as well as K_2_HPO_4_ and KH_2_PO_4_ for phosphate.

The optimisation results obtained through RSM revealed that the attained data are well-fitted with the model. The multiple correlation coefficients (*R*^*2*^) and *F* value were used to measure the strength of the model. According to Anderson and Whitcomb [62], a value of *R*^*2*^ near to 1.0 denotes a higher accuracy for the model. In this report, *R*^*2*^ values of 0.9446/0.9728 and adjusted *R*^*2*^ value of 0.8930/0.9475 for the respective ADL15/ADL36 strains pointed to the accuracy of the model, as it signifies a strong correlation between the experimental and predicted values. The accuracy of the model was also supported by a quite high *F* value of 18.28 and 38.35 for strains ADL15 and ADL36, respectively. Meanwhile, the insignificant lack of fit for strain ADL15 (*p* = 0.0502) and strain ADL36 (*p* = 0.0869) indicated that the acquired data was a good fit for the model [19]. This information showed that the response, which is based upon the dodecane mineralisation depends on both interacting and quadratic product of all tested parameters. Even though an abnormally high coefficient of variance (CV) at 29.13/16.77% obtained for each model of ADL15/ADL36 may depict a low precision and reliability in the overall response data, the adequate precision of 12.819/18.503 (ADL15/ADL36) that signifies the signal to noise ratio is desirable enough for the model to be used to navigate the design space.

Nevertheless, the extrapolation of dodecane or other hydrocarbon mineralisation under controlled laboratory scale to natural settings (e.g. Antarctic soil environments) proved to be a challenge due to the heightened degrees of complexity of actual environments, especially in the extreme conditions of Antarctica. The complexity of Antarctic soil environments arises from several factors which include the low soil temperature, range of different types and compositions of soil, physicochemical properties alterations by microenvironments, competition of consortia of different microorganism for the limited nutrient supply, and the occurrences of freeze–thaw cycle. Therefore, an understanding in both microbial growth and hydrocarbon degradation in Antarctic soil would enable environmental microbiologist in predicting the microbial response to anthropogenic perturbation of the environment (hydrocarbon pollution), microbial interaction with organic compound (hydrocarbon substrates), distribution of supplementary nutrients in soil and the survivability of microorganisms in the extreme and harsh environment.

## Conclusion

The optimisation of *n*-dodecane mineralisation from two Antarctic strain, ADL15 and ADL36 was reported by both conventional and statistical approaches. In the ‘one-factor-at-a-time’ method, both strains showed similar optimal condition for bacterial growth except for substrate concentration, where ADL36 showed higher tolerance towards diesel at 2% (v/v). Dodecane mineralisation by ADL36 also showed a higher degradation percentage at 83.75% than ADL15 (22.39%). The increase in dodecane mineralisation for the respective ADL15 (22.39–38.32%) and ADL36 (83.75–99.89%) by statistical response surface methodology proved that this approach is more suitable in predicting an optimal condition for a particular response. Optimisation by RSM also showed that it is more time and resource-positive methodology in providing a better comprehension of interactions that happened among the significant parameters that affect the dodecane mineralisation. From the study, strain ADL36 has been proved to be a better candidate than strain ADL15 for bioaugmentation operations on sites contaminated with aliphatic hydrocarbons especially in the Antarctic and other cold regions. Lastly, the dodecane degradation achieved by *Rhodococcus* sp. strain ADL36 hitherto can be interpreted as the highest reported by a single psychrotolerant strain to the best of our knowledge.

## Abbreviations

CCD: central composite design
OFAT: one-factor-at-a-time
PAHs: polycyclic aromatic hydrocarbons
PB: Plackett–Burman
RSM: response surface methodology
